# Risk prediction models for prolonged postoperative opioid use: a systematic review and meta-analysis of drug-risk stratification, validation and evidence benchmarks

**DOI:** 10.3389/fphar.2026.1946700

**Published:** 2026-10-14

**Authors:** Yin-sheng Liao, Ping Hou, Jian-li Song, Xuan Yu, Qiang Li, Bin Lu, Mao-yao Zheng, Guan-yu Chen

**Affiliations:** 1 Department of Anesthesiology, Zigong Fourth People’s Hospital, Zigong, Sichuan, China; 2 Department of Anesthesiology, Zigong First People’s Hospital, Zigong Academy of Medical Sciences, Zigong, Sichuan, China

**Keywords:** drug-risk stratification, opioid stewardship, prediction model, prolonged postoperative opioid use, systematic review

## Abstract

Prolonged postoperative opioid use represents a medication-safety problem in which short-term μ-opioid receptor agonist exposure may transition to sustained consumption, tolerance, opioid-induced hyperalgesia and dependence in susceptible patients. Risk prediction models could support clinical pharmacology-informed opioid stewardship by identifying patients who require individualised dosing, closer follow-up, alternative analgesic strategies or deprescribing support. We searched PubMed/MEDLINE, Embase, Web of Science, Scopus and the Cochrane Library from inception to 5 July 2026 for studies developing or validating multivariable models predicting opioid use beyond 30 days after surgery. One primary estimate was selected per independent cohort. Area under the receiver operating characteristic curve (AUROC) values were logit-transformed and pooled using random-effects restricted maximum likelihood models with Hartung-Knapp confidence intervals. 35 reports met the eligibility criteria and contributed 35 independent AUROC estimates to the quantitative synthesis. The pooled AUROC was 0.794 (95% CI 0.751–0.832), although between-study heterogeneity was extreme (I^2^ = 99%; 95% prediction interval, 0.512–0.934). Exploratory analyses suggested numerically lower discrimination in externally or temporally validated models and in opioid-naive cohorts, but these findings were not consistently supported by meta-regression. Current models identify a measurable drug-risk signal, yet few meet all four predefined translational-evidence benchmarks, and insufficient validation, calibration and clinical-utility evidence limits their immediate use for individualised opioid pharmacotherapy.

**Systematic Review Registration:**

https://www.crd.york.ac.uk/PROSPERO/view/CRD420261442043, identifier CRD420261442043.

## 1 Introduction

Opioid analgesics are central to the management of moderate-to-severe postoperative pain, but intended short-term use may persist beyond tissue recovery in susceptible patients, creating an important medication-safety problem. Prolonged use may coexist with persistent pain and increase the risk of opioid-related adverse outcomes and dependence, contributing to healthcare burden (Brummett et al., 2017). However, perioperative care often emphasises inpatient pain intensity and opioid consumption, while post-discharge medication trajectories receive less attention. Prediction models may help bridge this gap by identifying patients at risk of prolonged opioid use and guiding individualised follow-up and opioid-stewardship interventions.

The pharmacological basis for this transition is biologically plausible. Commonly used perioperative opioids act predominantly as μ-opioid receptor agonists. Repeated receptor activation can drive receptor phosphorylation, desensitisation, internalisation, tolerance and compensatory neuroadaptation (Pasternak and Pan, 2013; Williams et al., 2013). Longer exposure may also contribute to opioid-induced hyperalgesia and central sensitisation, in which escalating analgesic requirements coexist with worsening pain sensitivity (Angst and Clark, 2006; Colvin et al., 2019). At the behavioural and neurobiological level, sustained opioid exposure interacts with reward, withdrawal and reinforcement pathways that increase the risk of dependence and opioid use disorder in vulnerable patients (Volkow and McLellan, 2016). These mechanisms do not imply that every postoperative prescription is hazardous; they explain why duration, dose, formulation and follow-up matter.

The risk of new persistent postoperative opioid use varies substantially across individuals and surgical populations. Epidemiological evidence indicates that the incidence reaches approximately 5%–7% among adults undergoing general surgery and exceeds 10% following trauma, spinal and orthopaedic procedures (Brummett et al., 2017; Mohamadi et al., 2018; Lawal et al., 2020). Such heterogeneity is shaped by multiple clinical and contextual factors, including chronic pain, psychiatric comorbidities, substance use history, local prescribing practices and post-discharge follow-up accessibility (Sitter and Forget, 2021; Tay et al., 2022; Frangakis et al., 2026). Furthermore, interindividual differences in opioid response can be modulated by pharmacogenomic variability, particularly in genes governing drug metabolism, receptor function and pain modulation, such as cytochrome P450 2D6 (CYP2D6), the μ-opioid receptor gene (OPRM1) and the catechol-O-methyltransferase gene (COMT) (Crews et al., 2021). Accordingly, clinically meaningful prediction models should not be regarded as generic machine-learning classifiers, but rather as tools that may inform individualised decisions regarding opioid prescribing, monitoring and follow-up.

Previous systematic reviews have described rates and risk factors for prolonged postoperative opioid use, and broader reviews have summarised opioid-related prediction or machine-learning studies (Lawal et al., 2020; Mohamadi et al., 2018; Sitter and Forget, 2021). These syntheses established the clinical importance of the problem but did not quantify model discrimination while also evaluating validation design, calibration, decision curve analysis and risk of bias. For clinical pharmacology, this distinction is essential. A model that ranks patients well but gives poorly calibrated absolute risks cannot reliably guide opioid dosing or deprescribing decisions.

We therefore conducted a prospectively registered systematic review and meta-analysis of multivariable models developed or validated to predict opioid use beyond 30 days after surgery. The objectives were to quantify pooled discrimination, compare development/internal validation with external or temporal validation, explore sources of heterogeneity, summarise calibration and decision curve analysis reporting, and assess whether available models are sufficiently mature to support individualised opioid stewardship.

## 2 Methods

### 2.1 Protocol and reporting

This systematic review and meta-analysis of multivariable prediction models was designed, conducted and reported in accordance with the 2020 Preferred Reporting Items for Systematic Reviews and Meta-Analyses (PRISMA) statement, alongside the Transparent Reporting of a multivariable prediction model for Individual Prognosis Or Diagnosis extension for systematic reviews and meta-analyses of prediction models (TRIPOD-SRMA) (Snell et al., 2023; Page et al., 2021). Data extraction procedures followed the Critical Appraisal and Data Extraction for Systematic Reviews of Prediction Modelling Studies (CHARMS) checklist framework, and methodological quality appraisal utilised the Prediction model Risk Of Bias Assessment Tool (PROBAST) risk-of-bias tool for prediction model studies (Wolff et al., 2019; Moons et al., 2014). The full review protocol was prospectively registered in the International Prospective Register of Systematic Reviews (PROSPERO; registration number CRD420261442043).

### 2.2 Search strategy

PubMed/MEDLINE, Embase, Web of Science, Scopus and the Cochrane Library were searched from inception to 5 July 2026. Search terms combined concepts for surgery or peri-operative care, opioid analgesics, prolonged or persistent opioid use, and prediction modelling or machine learning. Procedure-specific terms were not used to avoid restricting the search to surgical areas already represented in the literature. The full database search strategies are provided in Supplementary Table S1.

All retrieved citations were exported to reference management software for automated deduplication followed by manual cross-checking to remove residual duplicate records. Two independent reviewers completed two-stage screening: initial title-and-abstract screening, followed by full-text assessment of all potentially eligible articles. Any discrepancies regarding eligibility were resolved via group discussion and re-evaluation of the full manuscript; persistent disagreements were adjudicated by a third senior reviewer. Final screening counts were summarised within a PRISMA flow diagram. Grey literature, conference abstracts without extractable full performance data, unpublished trial registries and narrative reviews were not eligible for inclusion.

### 2.3 Eligibility criteria

#### 2.3.1 Inclusion criteria

1. Original human studies enrolling patients undergoing any operative procedure;
2. Development, internal validation or external temporal/geographic validation of any multivariable predictive model for postoperative opioid use persisting beyond 30 days of surgery or discharge; eligible outcome labels included prolonged, persistent, sustained, chronic or long-term opioid consumption/prescription refill;
3. Multivariable modelling approaches permitted: conventional logistic regression, penalised regression, clinical risk scores/nomograms, tree-based machine learning, ensemble algorithms and externally validated pre-existing prediction tools;
4. Sufficient quantitative data available to extract or compute area under the receiver operating characteristic curve (AUROC) with associated uncertainty estimates for the target postoperative opioid outcome.

#### 2.3.2 Exclusion criteria

1. Studies reporting only univariable risk factors without a complete multivariable prediction model;
2. Predictive models for opioid-related outcomes unrelated to postoperative surgical populations;
3. Reviews, editorials, letters, conference abstracts lacking extractable AUROC statistics, and trial registration documents without primary empirical data;
4. Duplicate publications describing identical study cohorts and identical core predictive models (only the most complete, recently published full-text report was retained);
5. Studies without extractable AUROC values or corresponding confidence intervals/standard errors required for logit-scale pooling; such studies were excluded from both quantitative pooling and qualitative synthesis.

### 2.4 Data extraction

A standardised, pre-piloted data extraction form was developed, trialled on five randomly selected included manuscripts and refined prior to full-scale data abstraction. Two independent reviewers extracted data in duplicate, with all inconsistencies resolved by consensus without third-party adjudication where possible. Extracted variables fell into six broad domains: study metadata, population and surgical characteristics, baseline opioid exposure status, outcome definition and follow-up window, model development and validation design, and complete predictive performance metrics. Performance data extracted included AUROC with 95% confidence intervals (CIs), calibration metrics (calibration plots, calibration-in-the-large, calibration slope, intercept, Brier score), decision curve analysis (DCA) threshold ranges, sensitivity and specificity where reported, sample size and outcome event counts.

For manuscripts reporting multiple distinct prediction models derived from the same cohort, all available performance statistics were recorded in supplementary tables for qualitative reference. To avoid statistical non-independence and unit-of-analysis bias in the primary meta-analysis, only one primary performance estimate per unique study cohort was selected using a predefined hierarchical selection rule described below. Missing or ambiguous performance values were marked as “not reported”; standard error calculation was only attempted where raw event and non-event counts permitted application of the *Hanley–McNeil* method for AUROC variance estimation (Hanley and McNeil, 1982).

### 2.5 Primary model selection rule for meta-analysis

A hierarchical priority framework was applied to select a single representative AUROC estimate per independent cohort to avoid correlated multiple entries within the quantitative synthesis.

1. External or temporal geographic validation data (highest priority);
2. Split-sample or holdout internal validation;
3. Bootstrap/resampling internal validation;
4. Apparent performance derived solely from the model development dataset (lowest priority).

Within identical validation tiers, the model explicitly designated as the primary analytic tool by the study authors was selected. If no primary model was specified, the model demonstrating the highest validation-set AUROC was chosen, provided this choice did not contradict the authors’ own interpretive conclusions. An audit procedure was undertaken to quantify the magnitude of AUROC differences between the selected primary estimate and alternative candidate models within studies reporting multiple predictive algorithms, to assess whether the selection hierarchy systematically favoured artificially high discriminatory values. This audit was reported narratively without secondary quantitative pooling due to incomplete confidence interval data for alternative model variants.

### 2.6 Risk of bias and applicability assessment

All included primary model estimates underwent structured appraisal using the PROBAST tool, which evaluates risk of bias across four separate domains: participants, predictors, outcome and statistical analysis (Wolff et al., 2019). Each domain was rated low, unclear or high risk of bias; an identical three-tiered overall risk-of-bias grade was assigned to each independent model estimate. Separate PROBAST applicability judgements were documented as the level of concern regarding the applicability of the study population, predictor timing and outcome definition to the target clinical setting; judgements were classified as low, unclear or high concern. Two independent reviewers completed all PROBAST assessments, with disagreements resolved by consensus. Full domain-level ratings for each study are summarised in Supplementary Tables S4, S5.

### 2.7 Statistical analysis

#### 2.7.1 Primary pooling of AUROC

AUROC values were logit-transformed prior to meta-analytic pooling to satisfy normality assumptions for diagnostic test and predictive model performance synthesis. Standard errors for logit-AUROC were derived from published 95% confidence intervals where available. Where confidence intervals were absent but raw event/non-event counts were extractable, the *Hanley–McNeil* formula was applied to generate approximate standard errors solely for inclusion in the pooled model (Hanley and McNeil, 1982).

Summary AUROC estimates were calculated using random-effects restricted maximum likelihood (REML) models with Hartung–Knapp adjustment for confidence intervals to account for small numbers of included datasets (Snell et al., 2018). Pooled logit-scale values were back-transformed to the original AUROC scale for presentation. Between-study heterogeneity was quantified using I^2^ and the between-study variance tau^2^. The 95% prediction interval incorporated both sampling uncertainty and the estimated tau^2^ and was calculated to describe the expected range of true AUROC values in a comparable future clinical setting.

#### 2.7.2 Prespecified subgroup and sensitivity analyses

Predefined subgroup stratifications were conducted to explore sources of between-study heterogeneity, grouped by: validation status (external/temporal versus development/internal validation), baseline opioid exposure (opioid-naive versus mixed/prior opioid cohorts), prediction timing (preoperative, intraoperative/discharge, or postoperatively updated), surgical specialty domain, model class (conventional regression, machine-learning/penalised models, risk score/calculator/existing models, or other/unclear models), outcome follow-up window, operational definition of prolonged opioid use, completeness of calibration reporting, presence/absence of DCA, and overall PROBAST risk of bias (low, unclear, or high). Between-subgroup differences were tested using omnibus tests of interaction within the random-effects framework.

Two *a priori* sensitivity analyses were performed: exclusion of all model estimates rated high overall risk of bias by PROBAST; and exclusion of outlier studies reporting an unadjusted AUROC ≥0.95. Leave-one-out influence analysis was conducted to evaluate whether any single individual study exerted disproportionate influence on the overall pooled AUROC summary effect. All subgroup and sensitivity analyses were summarised in Tables 1, 2 with corresponding forest plots provided as supplementary figures.

**TABLE 1:** Primary and sensitivity analyses of AUROC.

Analysis	*n*	Pooled AUROC (95% CI)	95% prediction interval	I^2^	tau^2^	Model
Primary analysis	35	0.794 (0.751–0.832)	0.512–0.934	99	0.395	REML random effects with Hartung-Knapp CI
Excluding PROBAST high risk	26	0.787 (0.748–0.822)	0.552–0.917	99	0.272	REML random effects with Hartung-Knapp CI
Excluding AUROC ≥ 0.95	32	0.770 (0.742–0.797)	0.585–0.889	99	0.174	REML random effects with Hartung-Knapp CI

Abbreviations: AUROC, area under the receiver operating characteristic curve; CI, confidence interval; I^2^, proportion of total variability attributable to between-study heterogeneity; PROBAST, Prediction model Risk Of Bias Assessment Tool; REML, restricted maximum likelihood; tau^2^, estimated between-study variance. *n* denotes the number of independent study-level estimates included in each analysis.

**TABLE 2:** Prespecified exploratory subgroup meta-analyses of AUROC.

Subgroup domain	*n*	Pooled AUROC (95% CI)	95% prediction interval	I^2^	*P*
Validation level					0.572
Development only/unclear	8	0.811 (0.740–0.867)	0.551–0.938	99	
External validation	9	0.749 (0.697–0.795)	0.580–0.865	96	
Internal split/holdout validation	9	0.800 (0.696–0.875)	0.404–0.959	99	
Resampling/internal validation	9	0.834 (0.658–0.929)	0.214–0.989	96	
Validation status					0.170
Development/internal validation	26	0.811 (0.755–0.856)	0.480–0.952	99	
External/temporal validation	9	0.749 (0.697–0.795)	0.580–0.865	96	
Baseline opioid status					0.021
Mixed/prior use included	28	0.812 (0.764–0.853)	0.521–0.945	99	
Opioid-naive	7	0.713 (0.638–0.778)	0.498–0.862	89	
Prediction timing					0.325
Intraop/discharge/in-hospital	11	0.843 (0.723–0.916)	0.349–0.982	99	
Postoperative-updated	5	0.771 (0.677–0.843)	0.482–0.924	97	
Preoperative only	19	0.777 (0.723–0.824)	0.499–0.925	98	
Surgical/procedure domain					0.981
Mixed surgery	8	0.796 (0.728–0.851)	0.550–0.926	99	
Orthopaedic/extremity	14	0.794 (0.683–0.873)	0.346–0.966	95	
Spine/neurosurgery	7	0.800 (0.739–0.850)	0.601–0.914	89	
Other/non-orthopaedic	6	0.806 (0.612–0.917)	0.249–0.981	99	
Model class					0.246
Conventional regression	7	0.732 (0.659–0.794)	0.506–0.879	96	
Machine learning/penalized model	18	0.825 (0.742–0.885)	0.394–0.971	100	
Other/unclear	2	0.851 (0.435–0.977)	Not estimable	96	
Risk score/calculator/existing model	8	0.771 (0.738–0.801)	0.673–0.846	84	
Outcome-window					0.098
Early extended window: >30 days to <90 days	3	0.766 (0.733–0.797)	0.660–0.847	0	
Intermediate window: around 90–180 days	6	0.714 (0.657–0.765)	0.547–0.837	95	
Long window: ≥6 or 6–12 months	22	0.829 (0.768–0.877)	0.481–0.962	99	
Unclear/mixed follow-up window	4	0.733 (0.673–0.786)	0.558–0.857	82	
Operational outcome					0.683
Any fill/refill/use at follow-up definition	17	0.801 (0.712–0.868)	0.373–0.965	98	
Other or unclear operational definition	3	0.740 (0.414–0.920)	0.001–1.000	96	
Repeated or sustained prescription/supply definition	15	0.798 (0.756–0.835)	0.635–0.900	98	
Calibration reporting					0.014
Calibration not reported	13	0.856 (0.757–0.919)	0.377–0.983	99	
Calibration reported	22	0.759 (0.727–0.789)	0.589–0.874	98	
DCA reporting					0.125
DCA not reported	24	0.815 (0.755–0.863)	0.466–0.957	99	
DCA reported	11	0.751 (0.707–0.790)	0.581–0.867	98	
PROBAST overall					0.438
High	9	0.834 (0.658–0.929)	0.212–0.989	98	
Low	6	0.735 (0.691–0.775)	0.610–0.831	87	
Unclear	20	0.800 (0.751–0.840)	0.538–0.932	100	

Abbreviations: AUROC, area under the receiver operating characteristic curve; CI, confidence interval; DCA, decision curve analysis; PROBAST, Prediction model Risk Of Bias Assessment Tool. I^2^ denotes the proportion of total variability attributable to between-study heterogeneity, and n denotes the number of independent study-level estimates. *P* values are omnibus tests of between-subgroup differences.

Subgroup classifications were independently assigned at the study level according to the definitions reported in the original articles and were intended for exploratory interpretation. Validation level describes the four-category validation design: development only/unclear, internal split/holdout validation, resampling/internal validation and external validation. Validation status collapses these categories into development/internal validation versus external/temporal validation for the principal comparison.

Surgical/procedure domains were classified as orthopaedic/extremity surgery, including ACL, reconstruction, meniscal or other knee/hip arthroscopy, arthroplasty, hand or upper-extremity surgery, shoulder/rotator cuff surgery and pelvic or hip fracture surgery; spine/neurosurgery, including ACDF, lumbar disc or spine surgery and major spine surgery; mixed surgery, including heterogeneous surgical or mixed adolescent cohorts; or other/non-orthopaedic procedures, including cesarean delivery, other surgical cohorts and major elective surgery under general anesthesia.

Model class was assigned according to the primary model reported in Table 3: conventional regression, including multivariable logistic regression; machine-learning/penalised model, including gradient boosting, naive Bayes, bagging and other algorithmic or penalised approaches; risk score/calculator/existing model, including the SOS score and other previously developed or externally validated tools, such as SORG-MLA; or other/unclear model, including hybrid approaches or models with insufficient methodological detail. When multiple models were reported, classification followed the primary model selected for the meta-analysis.

Outcome-window categories were based on the reported follow-up period for prolonged postoperative opioid use, and operational-definition categories were based on whether opioid use was defined by any fill, refill or use; repeated or sustained prescription or supply; or another/unclear criterion.

#### 2.7.3 Exploratory univariable meta-regression

Separate univariable random-effects meta-regression models were fitted to quantify the study-level association of each predefined clinical and methodological covariate with logit-transformed AUROC. Covariates included all variables tested in subgroup analyses. To account for multiplicity, nominal omnibus *P* values from the 10 univariable meta-regression models were additionally adjusted using the Benjamini-Hochberg false-discovery-rate procedure. Both unadjusted P values and adjusted q values are reported, with the adjusted analyses interpreted as exploratory. All meta-regression analyses were summarised in Supplementary Table S3.

#### 2.7.4 Narrative synthesis of calibration and decision curve analysis

Quantitative pooling of calibration metrics and DCA net clinical benefit was not feasible due to extreme heterogeneity in reported indices, threshold values and graphical reporting formats across studies. All available calibration statistics (including median, interquartile range (IQR) and full range of extracted Brier scores) were collated and summarised descriptively, alongside qualitative review of DCA-derived net benefit curves and reported threshold windows.

#### 2.7.5 Composite framework of four predefined translational-evidence benchmarks

Because no universally accepted standard currently defines the clinical readiness of postoperative opioid-use prediction models, we applied a pragmatic, prespecified composite framework of four translational-evidence benchmarks covering four complementary dimensions: external or temporal validation as an indicator of transportability; formal calibration as an assessment of absolute-risk reliability; decision curve analysis as an indicator of potential net clinical benefit; and low overall PROBAST risk of bias as an indicator of methodological credibility. This pragmatic framework serves as transparent, clinically orientated evidence benchmarks and does not represent a validated or universally accepted definition of clinical readiness. Models meeting all four predefined translational-evidence benchmarks were classified as satisfying the full set of prespecified criteria; those meeting the first three criteria without low risk of bias were categorised as partially satisfying these benchmarks. Counts and percentages of estimates fulfilling each combination of benchmarks were tabulated in Supplementary Table S6 and visualised in Supplementary Figure S4.

#### 2.7.6 Software and statistical thresholds

All meta-analytical calculations, subgroup stratifications, meta-regression and sensitivity testing were performed using Stata 17.0 (StataCorp, College Station, TX, United States) with specialist prediction-model meta-analysis modules. A two-tailed statistical significance threshold of *P* < 0.05 was adopted for all inferential tests. Formal funnel plot-based evaluation of small-study effects and publication bias was not undertaken, as substantial between-study heterogeneity in model design, surgical cohorts and outcome definitions violated core assumptions of standard publication bias diagnostics for intervention meta-analyses.

## 3 Results

### 3.1 Study selection and baseline model characteristics

Initial database searches retrieved 2,938 citations, with 5 additional records identified through citation screening. After removal of 1,028 duplicates, 1,910 titles and abstracts were screened, and 209 full-text reports were assessed for eligibility. 35 reports met the eligibility criteria and were included in the review and quantitative synthesis (Gabriel et al., 2022; Karhade et al., 2020; Gessner et al., 2025; Tsai et al., 2023; Giladi et al., 2023; Lu et al., 2022; Castle et al., 2023; Kunze et al., 2021; Simpson et al., 2024; Nicholson et al., 2019; Soley et al., 2024; Harris et al., 2020; Ekman et al., 2026; Naderalvojoud and Hernandez-Boussard, 2023; Naderalvojoud et al., 2025; Bateman et al., 2016; Ward et al., 2021; Yeh et al., 2026; Sun et al., 2024; Chen et al., 2025; Singh et al., 2022; Chen et al., 2023; Chaudhary et al., 2019; Kim et al., 2025; Shipp et al., 2022; Crawford et al., 2023; Karhade et al., 2019; Zhang et al., 2020; Yen et al., 2022; Hur et al., 2021; Tudorache et al., 2026; Soley et al., 2025; Guattery et al., 2025; Anderson et al., 2020; Grazal et al., 2022). The remaining reports were excluded for the reasons shown in the PRISMA flow diagram (Figure 1).

**FIGURE 1:**
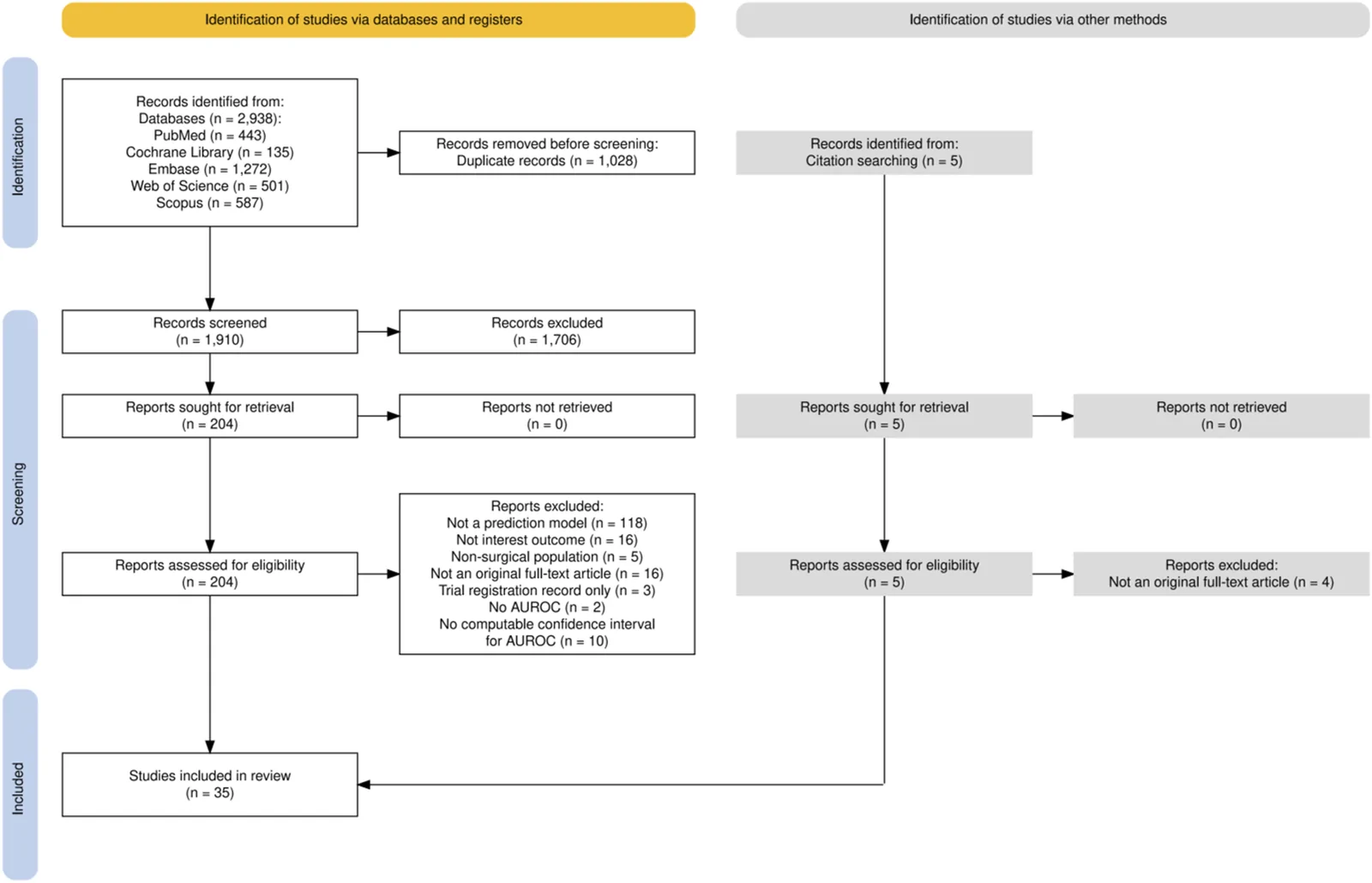
PRISMA flow diagram of study identification, screening, eligibility and inclusion.

The 35 study-level estimates represented heterogeneous surgical populations, prediction timings and modelling frameworks. Fourteen involved orthopaedic or extremity surgery, eight included mixed surgical populations, seven comprised spine or neurosurgical procedures, and six involved other non-orthopaedic procedures. Twenty-eight analyses included patients with prior or concurrent opioid exposure, whereas seven exclusively enrolled opioid-naive individuals. Prediction was based exclusively on preoperative information in 19 analyses, incorporated intraoperative, in-hospital or discharge data in 11, and was updated using postoperative information in 8. Eighteen primary models used machine-learning or penalised-regression methods, eight evaluated named risk scores, calculators or existing models, seven used conventional multivariable regression, and two involved other or insufficiently specified modelling approaches. Nine estimates underwent external or temporal validation, nine used split-sample internal validation, nine used resampling-based internal validation, and eight were based on development-only analyses or had an unclear validation design (Table 3).

**TABLE 3:** Study and model characteristics.

Study	Country	Surgical population	Baseline opioid status	N/events	Outcome window	Prediction timing	Primary model	Validation level	AUROC (95% CI)	Cal/DCA	PROBAST
Anderson AB 2020	US	ACL reconstruction	Mixed/prior use included	10,919/NR	Prolonged use: any opioid Rx > 90 days after ACL reconstruction (90–365 days follow-up)	Preoperative only	Gradient boosting machine	Internal split/holdout validation	0.770 (0.750–0.800)	Yes/Yes	Low
Bateman BT 2016	US	Cesarean delivery	Opioid-naive	80,127/285	Persistent use: trajectory-defined monthly opioid filling during 12 months after cesarean delivery	Intraop/discharge/in-hospital	Multivariable logistic regression/trajectory-based persistent-use prediction model	Resampling/internal validation	0.670 (0.635–0.704)	No/No	High
Castle 2023	US	Meniscal surgery	Mixed/prior use included	581/98	Prolonged use: opioid refill ≥ 1 month after arthroscopic meniscus surgery	Intraop/discharge/in-hospital	Naive Bayes model	Development only/unclear	0.790 (0.670–0.875)	Yes/No	High
Chaudhary MA 2019	US	Mixed surgery	Mixed/prior use included	86,356/6,365	Chronic postoperative opioid use: uninterrupted opioid Rx for ≥ 6 months post-op	Intraop/discharge/in-hospital	Stopping opioids after surgery (SOS) score	Internal split/holdout validation	0.760 (0.746–0.774)	Yes/No	High
Chen 2025	Taiwan	ACDF	Mixed/prior use included	1,037/34	Sustained Rx: continuous opioid use ≥ 90 days after ACDF	Preoperative only	SORG-MLA	External validation	0.780 (0.700–0.870)	Yes/Yes	Low
Chen SF 2023	Taiwan	Lumbar spine surgery	Opioid-naive	2,795/146	Prolonged opioid prescription: continuous opioid Rx 90–180 days after lumbar spine surgery	Preoperative only	SORG-MLA	External validation	0.710 (0.660–0.750)	Yes/Yes	Low
Crawford AM 2023	US	Mixed surgery	Mixed/prior use included	26,114/1,039	Sustained opioid prescriptions: continuous opioid use ≥ 90 days post-op	Intraop/discharge/in-hospital	Stopping opioids after surgery (SOS) score	External validation	0.820 (0.804–0.835)	No/No	Unclear
Ekman E 2026	Finland	Pelvic fracture surgery	Mixed/prior use included	233/94	Prolonged use: ≥ 1 opioid Rx after post-op month 3 (assessed at 3–12 months)	Preoperative only	Multivariable logistic regression model	Development only/unclear	0.690 (0.615–0.756)	Yes/No	Unclear
Gabriel 2022	US	LE arthroplasty	Mixed/prior use included	1,042/242	Persistent use: continued opioid use 3–6 months after arthroplasty	Intraop/discharge/in-hospital	Balanced bagging classifier	Resampling/internal validation	0.959 (0.896–0.985)	No/No	High
Gessner KH 2025	US	Mixed surgery	Opioid-naive	4,116/245	New persistent use: ≥ 1 opioid Rx 90–180 days after surgery in opioid-naive patients	Preoperative only	Model E + C + G logistic regression	Resampling/internal validation	0.667 (0.619–0.717)	Yes/No	Unclear
Giladi 2023	US	Hand/UE surgery	Mixed/prior use included	1,656/142	Persistent use: continued opioid use at 6 months after surgery	Preoperative only	XGBoost model	Resampling/internal validation	0.730 (0.660–0.790)	Yes/No	Unclear
Grazal 2022	US	Arthroscopic hip surgery	Mixed/prior use included	6,760/2,762	Prolonged postoperative opioid use: opioid fill between 90 and 365 days after hip arthroscopy	Postoperative-updated	Artificial neural network model	Development only/unclear	0.710 (0.680–0.740)	Yes/Yes	Unclear
Guattery J 2025	US	Rotator cuff repair	Mixed/prior use included	852/172	Prolonged postoperative opioid use: opioid prescription fill ≥ 90 days after rotator cuff surgery	Intraop/discharge/in-hospital	XGBoost model	Resampling/internal validation	0.990 (0.972–0.997)	No/No	High
Harris 2020	US	ACDF	Mixed/prior use included	28,813/4,986	Chronic postoperative opioid use: ≥ 120 days supply or ≥ 10 opioid prescriptions between months 3 and 12	Preoperative only	Final multivariable logistic regression model	Development only/unclear	0.770 (0.762–0.778)	No/No	High
Hur 2021	US	Other surgery	Opioid-naive	112,989/6,531	New persistent use: opioid fill 91–180 days after surgery in opioid-naive patients	Preoperative only	XGBoost model for new persistent use	Internal split/holdout validation	0.660 (0.640–0.680)	No/No	High
Karhade 2019	US	Mixed surgery	Mixed/prior use included	7,027/604	Sustained prescription opioid use: opioid prescription without interruption through 90–180 days post-op	Intraop/discharge/in-hospital	Stopping opioids after surgery (SOS) score	External validation	0.764 (0.741–0.786)	No/No	Unclear
Karhade AV 2020	US	Lumbar spine surgery	Mixed/prior use included	4,165/61	Sustained postoperative prescription opioid exposure at 6 months after lumbar surgery	Intraop/discharge/in-hospital	Stopping opioids after surgery (SOS) score	External validation	0.820 (0.740–0.870)	No/No	Unclear
Kim J 2025	South Korea	Mixed surgery	Mixed/prior use included	1108119/9,308	Chronic opioid use: ≥ 10 prescriptions or >120 days supply between 91 and 365 days after discharge	Intraop/discharge/in-hospital	Gradient boosting machine	Development only/unclear	0.875 (0.871–0.879)	No/No	Unclear
Kunze 2021	US	Hip arthroscopy	Opioid-naive	775/227	Prolonged use: any opioid use beyond 1 month after hip arthroscopy in opioid-naive patients	Preoperative only	Stochastic gradient boosting	Internal split/holdout validation	0.750 (0.630–0.830)	Yes/Yes	Unclear
Lu Y 2022	US	Knee arthroscopy	Mixed/prior use included	381/60	Long-term opioid use: filling ≥ 180-day supply of opioids within 1 year after surgery	Postoperative-updated	Complete ensemble model	Resampling/internal validation	0.740 (0.658–0.808)	Yes/Yes	Unclear
Naderalvojoud 2025	US	Other surgery	Mixed/prior use included	397,150/136,222	Prolonged use: opioid exposure between 90 and 180 days after surgery (model validation/fairness analysis)	Preoperative only	Lasso logistic regression	External validation	0.700 (0.698–0.702)	Yes/Yes	Unclear
Naderalvojoud 2023	US	Other surgery	Mixed/prior use included	41,675/6,071	Prolonged use: any opioid exposure 90–180 days after surgery	Preoperative only	Ensemble lasso logistic regression	Resampling/internal validation	0.784 (0.769–0.800)	Yes/No	Unclear
Nicholson AD 2019	US	Shoulder surgery	Mixed/prior use included	548/153	Prolonged use: opioid use >6 weeks after shoulder surgery	Preoperative only	Restricted logistic regression model	Resampling/internal validation	0.766 (0.736–0.820)	Yes/No	Unclear
Shipp MM 2022	US	Hand/UE surgery	Mixed/prior use included	2,114/212	Prolonged use: opioid refill/use at 3 months after upper extremity surgery	Preoperative only	Main lasso/multivariable model using EHR and PRD	External validation	0.603 (0.513–0.686)	Yes/No	Unclear
Simpson S 2024	US	Major spine surgery	Mixed/prior use included	2,611/1,209	Persistent use: opioid use at 3 months after major spine surgery	Preoperative only	Balanced random forest classifier with SMOTE	Internal split/holdout validation	0.877 (0.834–0.894)	Yes/No	Unclear
Singh K 2022	US	Mixed surgery	Mixed/prior use included	24,040/4,879	Persistent use: filling opioids in days 4–90 and 91–180 after surgery	Preoperative only	Full prediction model	Internal split/holdout validation	0.870 (0.860–0.880)	Yes/Yes	Unclear
Soley 2024	US	Other surgery	Mixed/prior use included	347/42	Chronic opioid use: ≥ 1 opioid prescription filled between 6 and 12 months after surgery	Preoperative only	Stacking ensemble model	Development only/unclear	0.900 (0.880–0.920)	No/No	Unclear
Soley 2025	US	Other surgery	Mixed/prior use included	271/32	Chronic opioid use: ≥ 1 opioid prescription filled between 6 and 12 months after surgery	Preoperative only	Stacking ensemble model with genetic, EHR, and wearable data	Internal split/holdout validation	0.950 (0.890–0.950)	No/No	Unclear
Sun M 2024	Taiwan	Major elective surgery under general anesthesia	Mixed/prior use included	111,069/1812	Chronic opioid use: opioid use >90 days after surgery	Intraop/discharge/in-hospital	Wu and Zhang predictive scoring system	Resampling/internal validation	0.830 (0.818–0.841)	No/No	High
Tsai CC 2023	Taiwan	Total knee arthroplasty	Mixed/prior use included	3,495/114	Prolonged postoperative opioid prescription: continuous opioid use after TKA (original SORG definition)	Preoperative only	SORG-MLA	External validation	0.750 (0.700–0.810)	Yes/Yes	Low
Tudorache Ym 2026	Denmark	Hip fracture surgery	Opioid-naive	26,057/8,095	Long-term opioid therapy: Redeeming ≥ 2 opioid prescriptions between 31 and 365 days after hip fracture surgery	Intraop/discharge/in-hospital	Backward-elimination logistic regression model	Internal split/holdout validation	0.680 (0.660–0.690)	Yes/Yes	Low
Ward A 2021	US	Mixed adolescent surgery	Mixed/prior use included	186,493/8,410	Prolonged opioid use after surgery: opioid use during postoperative follow-up	Postoperative-updated	Day-of-surgery gradient boosting machine	Internal split/holdout validation	0.711 (0.699–0.723)	No/No	High
Yeh Ph 2026	Taiwan	TKA	Mixed/prior use included	12,958/1,029	Long-term opioid use: repeated opioid prescriptions ≥ 2 times between 90 and 360 days after TKA	Postoperative-updated	LASSO-elastic net penalized logistic regression	Development only/unclear	0.821 (0.808–0.835)	Yes/No	Unclear
Yen 2022	Taiwan	Lumbar disc surgery	Mixed/prior use included	1,316/41	Prolonged postoperative opioid prescription: continuous opioid use to at least 90–180 days after lumbar disc herniatio	Preoperative only	SORG-MLA	External validation	0.760 (0.690–0.830)	Yes/Yes	Low
Zhang Y 2020	US	Lumbar spine surgery	Opioid-naive	19,317/903	Long-term opioid use: filling ≥ 180-day supply of opioids within 1 year after surgery	Postoperative-updated	Full logistic regression model	Development only/unclear	0.847 (0.808–0.879)	Yes/No	Unclear

Abbreviations: ACL, anterior cruciate ligament; ACDF, anterior cervical discectomy and fusion; AUROC, area under the receiver operating characteristic curve; Cal, calibration; CI, confidence interval; DCA, decision curve analysis; d, days; E + C + G, electronic health record plus claims and geographic factors; EHR, electronic health record; LE, lower extremity; LASSO, least absolute shrinkage and selection operator; ML, machine learning; MME, morphine milligram equivalents; mo, months; NR, not reported; post-op, postoperative; PRD, patient-reported data; PRO, patient-reported outcome; PROBAST, Prediction model Risk Of Bias Assessment Tool; Rx, prescription; SMOTE, synthetic minority oversampling technique; SOS, Stopping Opioids after Surgery; SORG-MLA, Skeletal Oncology Research Group machine-learning algorithm; TKA, total knee arthroplasty; UE, upper extremity; US, United States; wk, weeks; XGBoost, extreme gradient boosting; y, years. N/events denotes the total sample size and number of outcome events, respectively.

The models varied from concise clinical scores to high-dimensional electronic health record (EHR) or claims-based algorithms. Commonly retained predictor families included previous opioid exposure or opioid dose, age, sex, mental health diagnoses, substance use, pain diagnoses, comorbidity, benzodiazepine or antidepressant exposure, procedure characteristics, prescription features and patient-reported outcomes (Supplementary Table S2). Several recent studies extended the predictor space to patient-reported data, multimodal EHR and claims data, genetic or wearable variables, and fairness-aware modelling. This breadth reflects the complexity of postoperative opioid use but also complicates direct comparison between models.

### 3.2 Primary meta-analysis of discriminatory performance (AUROC)

Across the 35 independent primary estimates, the pooled AUROC for prolonged postoperative opioid use was 0.794 (95% CI 0.751–0.832). Between-study heterogeneity was extreme (I^2^ = 99%; tau^2^ = 0.395), and the 95% prediction interval was 0.512–0.934. The 95% CI quantifies uncertainty around the pooled mean, whereas the prediction interval additionally incorporates between-study heterogeneity and describes the range in which the true AUROC of a comparable future setting may lie (Figure 2; Table 1).

**FIGURE 2:**
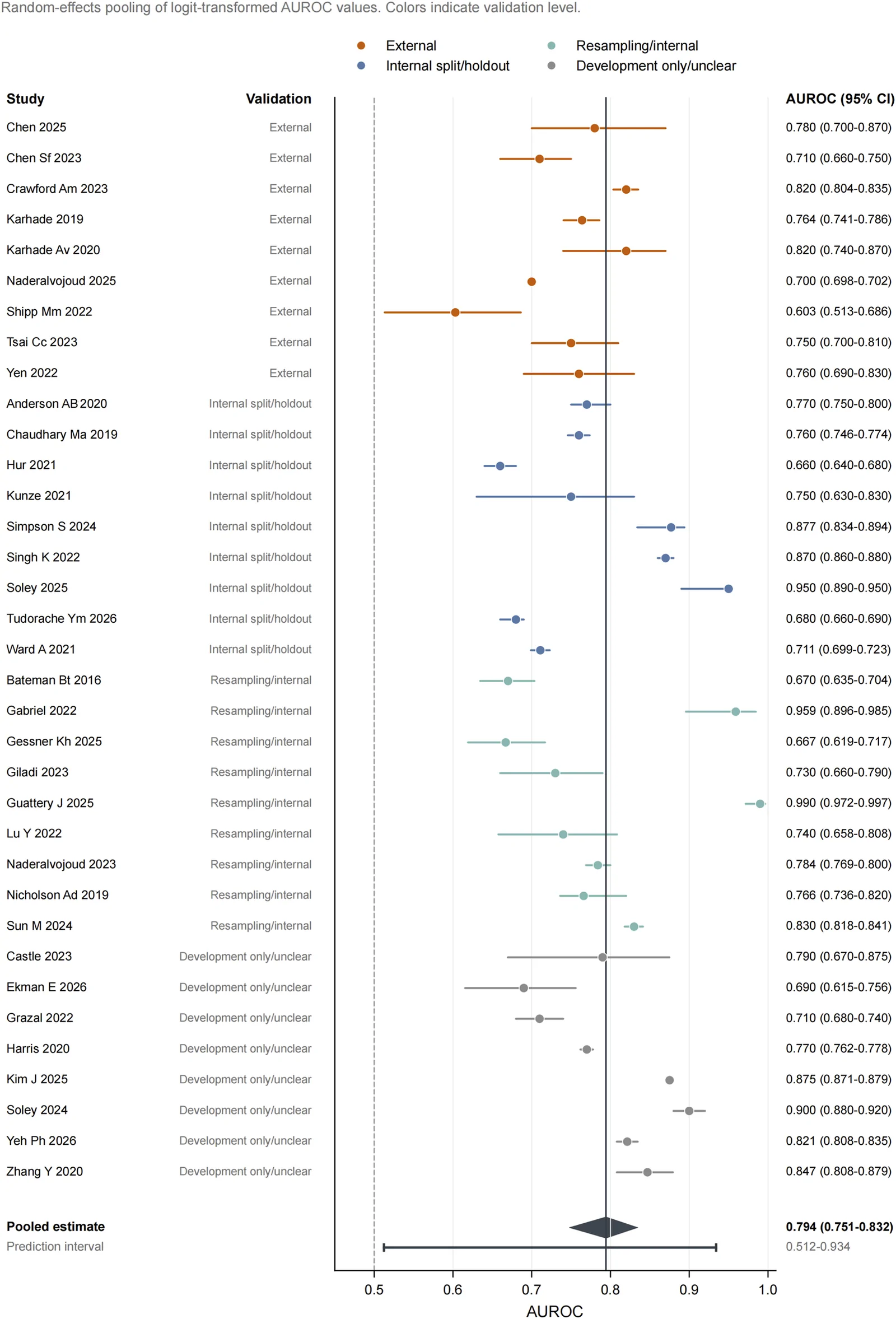
Forest plot of primary AUROC estimates from the 35 independent study-level model contexts included in the quantitative synthesis.

### 3.3 Exploratory sensitivity analysis

The primary estimate was robust to exclusion of the nine estimates rated high risk of bias by PROBAST (AUROC 0.787, 95% CI 0.748–0.822; 95% prediction interval 0.552–0.917) and to exclusion of the three estimates with AUROC ≥0.95 (AUROC 0.770, 95% CI 0.742–0.797; 95% prediction interval 0.585–0.889). The three high-AUROC estimates were machine-learning models assessed only in derivation or internal-validation settings (Figure 3; Table 1). Leave-one-out analysis did not identify a single estimate that materially changed the pooled result, and heterogeneity remained high throughout (Supplementary Figure S1).

**FIGURE 3:**
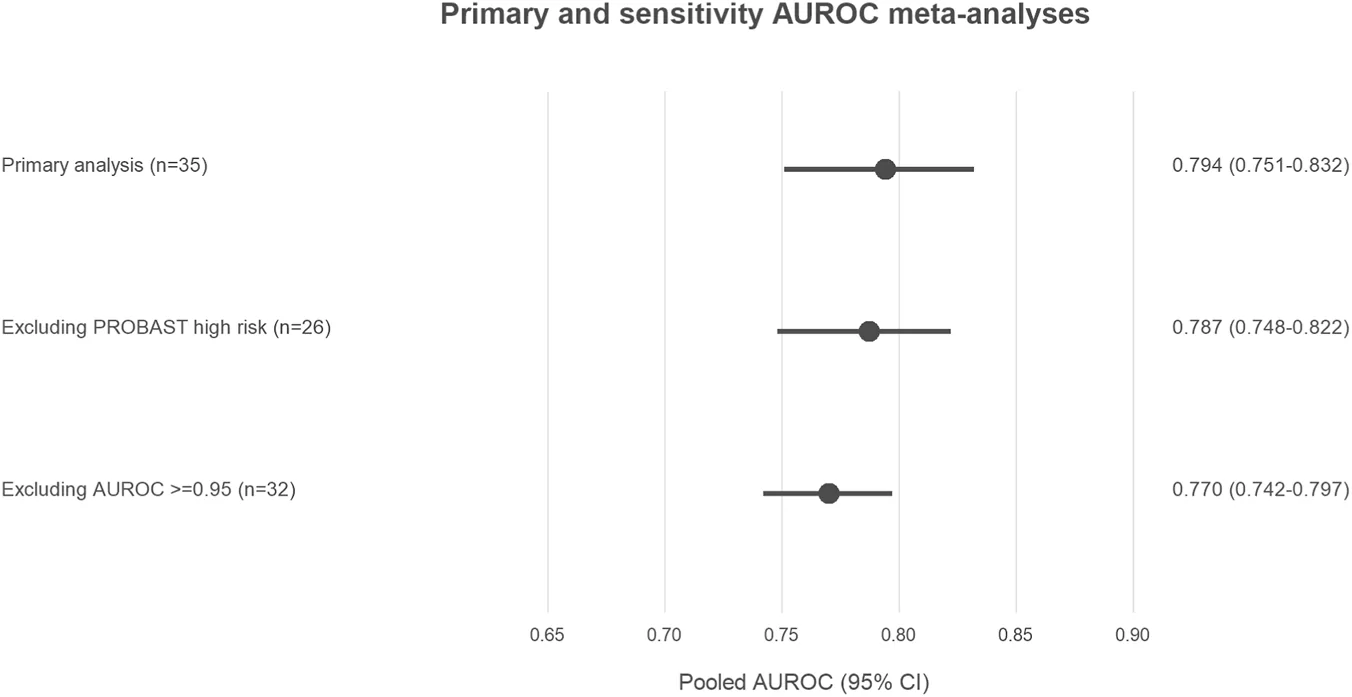
Primary and sensitivity AUROC meta-analyses.

### 3.4 Prespecified exploratory subgroup analyses

Across the prespecified exploratory subgroup analyses, pooled discrimination was numerically lower in externally or temporally validated models than in development or internally validated models (AUROC 0.749 vs. 0.811; subgroup *P* = 0.170). Nominal subgroup differences were observed according to baseline opioid status, with lower discrimination in opioid-naive than in mixed or prior-use cohorts (0.713 vs. 0.812; *P* = 0.021), and according to calibration reporting, with lower pooled AUROC among studies reporting calibration than among those not reporting it (0.759 vs. 0.856; *P* = 0.014). No clear subgroup differences were identified for prediction timing, surgical domain, model class, outcome window, operational outcome definition, DCA reporting or overall PROBAST risk (all subgroup *P* = 0.098–0.981; Table 2; Supplementary Figure S2).

### 3.5 Exploratory univariable meta-regression

In exploratory univariable meta-regression, calibration reporting was the only nominally significant moderator of AUROC (beta = −0.549 on the logit scale, 95% CI −1.025 to −0.072; omnibus *P* = 0.025). Baseline opioid status showed a non-significant trend (omnibus *P* = 0.072), and validation status, prediction timing, surgical domain, model class, outcome window, operational outcome definition, DCA reporting and PROBAST overall risk were not significant moderators (all omnibus *P* ≥ 0.156). No moderator remained significant after false-discovery-rate adjustment. These study-level analyses were exploratory and do not establish causal or independent effects (Supplementary Table S3).

### 3.6 PROBAST risk-of-bias and applicability assessment

PROBAST appraisal classified 6 of the 35 primary estimates as low overall risk of bias, 20 as unclear risk and 9 as high risk (Supplementary Figure S3; Supplementary Tables S4, S5). The recurring concerns were reliance on derivation or internal validation without external testing, unclear handling of missing predictor or outcome data, incomplete calibration assessment, low event counts relative to model complexity and selective reporting of model variants. Applicability concerns were generally low; these judgements addressed the relevance of the populations, predictor timing and outcome definitions to the target clinical setting rather than discriminatory performance.

### 3.7 Exploratory reporting-completeness analysis

Calibration metrics were available for 22 of the 35 primary estimates, whereas decision curve analysis (DCA) was reported for 11. Calibration outputs included calibration plots, calibration-in-the-large, calibration slopes or intercepts, goodness-of-fit tests and Brier scores. Reported Brier scores had a median of 0.078 (IQR 0.043–0.132; range 0.028–0.240), indicating substantial variation in overall probabilistic prediction error. DCA threshold definitions and reporting formats varied considerably, precluding quantitative pooling of net clinical benefit.

Applying this pragmatic framework (Supplementary Figure S4; Supplementary Table S6), five estimates (14.3%) satisfied three core translational benchmarks (external validation + calibration + DCA) (Tsai et al., 2023; Naderalvojoud et al., 2025; Chen et al., 2025; Chen et al., 2023; Yen et al., 2022), and four datasets (11.4%) fulfilled all four criteria simultaneously (Tsai et al., 2023; Chen et al., 2025; Chen et al., 2023; Yen et al., 2022). Most internally derived or internally validated models demonstrated moderate-to-high AUROC values but lacked at least one metric needed to judge real-world clinical utility, most commonly external validation, formal calibration or DCA-derived net benefit.

## 4 Discussion

This review reframes prolonged postoperative opioid-use prediction as a problem of drug-risk stratification. The pooled AUROC of 0.794 demonstrates that published models capture a clinically measurable risk signal. From a pharmacological perspective, the more critical finding is that this signal is not sufficiently robust to support individualised opioid therapy in routine clinical practice. Model performance varied substantially across study populations and validation settings; calibration was frequently incomplete, and only a small proportion of model estimates met all four predefined translational-evidence benchmarks.

The present meta-analysis revealed extremely high between-study heterogeneity, indicating that the pooled predictive accuracy cannot represent a generalisable performance of models for predicting prolonged postoperative opioid use. Such substantial heterogeneity stems from inconsistent predictive settings across primary studies, which vary widely in baseline opioid exposure profiles, surgical populations, outcome definitions, follow-up durations, prediction time points, variable availability, and validation methodologies.

Baseline opioid exposure constitutes a pivotal source of heterogeneity and should not be treated merely as a conventional covariate, as it defines two fundamentally distinct predictive tasks. For patients with prior opioid exposure, medication history acts as a robust risk marker that reflects drug tolerance, chronic pain conditions, and established prescribing patterns. In contrast, prediction for opioid-naive patients focuses on identifying the transition from intentional short-term postoperative analgesia to sustained opioid consumption. This process is modulated by initial postoperative prescriptions and relies on inconsistently documented clinical variables, rendering prediction inherently more challenging (Shah et al., 2017). Despite these conceptual distinctions, statistical evidence for performance differences across populations remains weak. Subgroup analyses showed numerically higher discrimination among mixed cohorts including opioid-exposed patients compared with opioid-naive cohorts (subgroup *P* = 0.021). However, this subgroup difference was not supported by univariable meta-regression (omnibus *P* = 0.072), nor did it persist following false-discovery-rate adjustment. Separate development and validation strategies may still merit consideration for opioid-exposed versus opioid-naive populations.

Model discrimination was also associated with calibration reporting. Studies reporting calibration had a lower pooled AUROC, and calibration reporting was the only nominally significant moderator in the univariable meta-regression (omnibus *P* = 0.025). This association should not be interpreted as evidence that calibration assessment reduces discrimination. It may instead reflect differences in study design, validation quality, reporting completeness or selective reporting of favourable performance estimates. After false-discovery-rate adjustment, no moderator remained statistically significant. These findings are therefore hypothesis-generating rather than confirmatory and reinforce that pooled AUROC alone is insufficient to establish the generalisability or clinical utility of postoperative opioid-use prediction models.

Sensitivity and validation analyses showed numerical differences suggestive of potential optimistic bias in reported model performance, though this between-group difference was not statistically significant. Excluding studies at high overall PROBAST risk of bias slightly reduced pooled AUROC to 0.787, while removing models with AUROC ≥0.95 yielded a pooled estimate of 0.770. Models from external or temporal validation demonstrated numerically lower discrimination than those from development or internal-validation datasets. While this pattern aligns with performance attenuation when models are applied to new populations, it does not constitute definitive evidence of optimistic bias in the present dataset (Siontis et al., 2015). Therefore, before models are used to guide clinical prescribing, external validation should be paired with rigorous calibration, alongside local model updating or recalibration where needed.

Drawing on the principles of the TRIPOD statement and PROBAST framework, we prespecified four pragmatic benchmarks to provide a transparent and reproducible map of translational evidence: external validation, calibration, decision-curve analysis and low overall PROBAST risk of bias. These benchmarks represent complementary indicators of model transportability, absolute-risk reliability, potential net benefit and methodological credibility, but were not intended as gold standards or a universal definition of clinical readiness. Other important considerations include adequate validation sample sizes and event counts, quantification of uncertainty in individual-level predictions, performance across clinically relevant patient subgroups, model updating or recalibration, and assessment of overfitting and optimism. For machine-learning models, transparent reporting of predictor variables, data preprocessing, missing-data handling and model outputs is also essential (Bouwmeester et al., 2012; Steyerberg et al., 2010). These additional features could not be incorporated into the composite framework because they were reported inconsistently and incompletely across the included studies.

Satisfying the four benchmarks only meets minimum methodological requirements and does not equate to established clinical-pharmacological decision-making value. Clinical implementation requires risk thresholds to be linked to clear, safe management strategies. Standardised short-course regimens plus routine follow-up may be applied for low-risk patients. A high-risk signal should not prompt automatic opioid dose reduction or withholding of analgesia; instead, it should trigger comprehensive pain and medication assessment, multimodal pharmacological and non-pharmacological analgesia, prescription review, early follow-up and specialist referral when indicated (Wetzel et al., 2018; Burns et al., 2021). A single-centre observational study suggested that intrathecal morphine combined with intrathecal magnesium, structured non-opioid multimodal analgesia and selective reduction of conventional systemic opioid exposure was associated with reduced early postoperative opioid use (Williams et al., 2025). Drug dose, formulation and treatment duration should be dynamically adjusted according to pain severity, functional recovery, adverse effects, prior opioid exposure and patient preferences. Model-guided management pathways require prospective validation to confirm reductions in prolonged opioid use without exacerbating inadequate analgesia, adverse events or impaired functional recovery.

Prediction models can do more than distinguish patients according to medication-related risk; they can also link high-risk patient characteristics to modifiable clinical care processes. In patients with prior opioid exposure, preoperative medication reconciliation and analgesic planning may help avoid unnecessary escalation of postoperative opioid doses. Initial prescription dose, early refill requests, gaps in pain follow-up and unaddressed psychological or substance-use problems represent potential opportunities for targeted intervention. In addition, perioperative opioid exposure should not be characterised solely by total morphine milligram equivalents. Opioid type, formulation, route of administration and duration of exposure may have different pharmacological effects and clinical consequences. Future models should evaluate whether distinguishing potentially higher-abuse-liability opioid exposure from other opioid strategies provides incremental predictive value beyond baseline opioid exposure history and total opioid dose. Notably, these factors represent only potential intervention targets, and prospective studies of patient-centred outcomes need to assess their clinical effects.

Machine-learning or penalised models showed numerically higher discrimination than conventional regression models in this review, but the difference was not statistically significant and heterogeneity within the machine-learning group was extreme (I^2^ = 100%). Thus, the available evidence does not demonstrate that greater algorithmic complexity provides a clinically meaningful advantage. Models with limited transparency may also be more difficult to audit, reproduce and integrate into prescribing workflows, even when their apparent AUROC is high. When simpler and more interpretable models provide comparable performance, they may be preferable for routine implementation. Explainability tools can support interpretation, but they cannot substitute for external validation, calibration or prospective clinical-impact evaluation (Christodoulou et al., 2019).

This review has several limitations. There was substantial heterogeneity across included studies regarding surgical populations, definitions of opioid use, follow-up windows, predictors and validation designs. All subgroup analyses, sensitivity analyses and univariable meta-regression performed in this study were exploratory in nature without pre-specified statistical power. Calibration and decision-curve analysis could not be pooled due to incomplete and inconsistent reporting. Ten potentially relevant full-text reports met the clinical and modelling eligibility criteria but lacked sufficient AUROC information. According to our prespecified exclusion criteria, these 10 studies were excluded from quantitative meta-analysis, qualitative synthesis and evaluation against the four predefined translational-evidence benchmarks, thereby limiting the completeness of the evidence assessment. Furthermore, most cohorts were recruited from North American healthcare systems, which limits generalisability to settings with different opioid-related regulations, prescribing norms and analgesic follow-up accessibility.

Future work should prioritise external and temporal validation of existing models, with local recalibration and stratified assessment for opioid-naive and opioid-exposed patients. It should also incorporate formulation-based drug risk stratification to compare the predictive value of opioids with high versus low abuse-liability profiles. Prospective impact studies are required to test whether model-guided care reduces prolonged opioid use while preserving pain control, function and patient satisfaction.

## 5 Conclusion

Existing prediction models for prolonged postoperative opioid use achieve moderate discrimination but seldom meet all four predefined translational-evidence benchmarks. Numerical differences in discrimination have been observed between opioid-naive and opioid-exposed populations, though their robustness remains uncertain. Possible optimistic bias, imperfect calibration and variable model transparency may hinder routine clinical implementation. These models may facilitate risk-stratified opioid stewardship and targeted follow-up, yet they are unsuitable for standalone individual prescribing decisions. Further external validation, recalibration and prospective impact assessment are necessary prior to clinical deployment.

## Data Availability

The original contributions presented in the study are included in the article/Supplementary Material, further inquiries can be directed to the corresponding authors.

## Supplementary material

SUPPLEMENTARY FIGURE S1 Leave-one-out influence analysis for the primary AUROC meta-analysis.

SUPPLEMENTARY FIGURE S2 Exploratory subgroup AUROC meta-analyses.

SUPPLEMENTARY FIGURE S3 PROBAST risk-of-bias and applicability concern categories.

SUPPLEMENTARY FIGURE S4 Evidence and reporting matrix for the 35 primary AUROC estimates.
