# Supplementary material for: Risk prediction models for prolonged postoperative opioid use: a systematic review and meta-analysis of drug-risk stratification, validation and evidence benchmarks
Source: Front Pharmacol. 2026 Oct 14;17:1946700. doi: 10.3389/fphar.2026.1946700 (PMC13713708; doi:10.3389/fphar.2026.1946700)
Supplement: Supplementary file 1 [file Supplementaryfile1.docx]

# Table S1. Full database search strategies.

1.PubMed

| **Search number** | **Query** | **Results** |
| --- | --- | --- |
| #1 | "Surgical Procedures, Operative"[Mesh] OR "Postoperative Period"[Mesh] OR "Perioperative Period"[Mesh] OR surgery[Title/Abstract] OR surgical[Title/Abstract] OR surgeries[Title/Abstract] OR operative[Title/Abstract] OR operation[Title/Abstract] OR operations[Title/Abstract] OR postoperative[Title/Abstract] OR post-operative[Title/Abstract] OR postsurgical[Title/Abstract] OR post-surgical[Title/Abstract] OR perioperative[Title/Abstract] OR peri-operative[Title/Abstract] OR "after surgery"[Title/Abstract] OR "following surgery"[Title/Abstract] | 5617440 |
| #2 | "Analgesics, Opioid"[Mesh] OR opioid*[Title/Abstract] OR opiate*[Title/Abstract] OR narcotic*[Title/Abstract] OR oxycodone[Title/Abstract] OR hydrocodone[Title/Abstract] OR morphine[Title/Abstract] OR codeine[Title/Abstract] OR tramadol[Title/Abstract] OR fentanyl[Title/Abstract] OR buprenorphine[Title/Abstract] | 239647 |
| #3 | "persistent postoperative opioid use"[Title/Abstract] OR "persistent post-operative opioid use"[Title/Abstract] OR "persistent postsurgical opioid use"[Title/Abstract] OR "persistent post-surgical opioid use"[Title/Abstract] OR "prolonged postoperative opioid use"[Title/Abstract] OR "prolonged post-operative opioid use"[Title/Abstract] OR "prolonged postsurgical opioid use"[Title/Abstract] OR "prolonged postoperative opioid prescription"[Title/Abstract] OR "prolonged postoperative opioid prescriptions"[Title/Abstract] OR "sustained postoperative opioid use"[Title/Abstract] OR "sustained postoperative opioid prescription"[Title/Abstract] OR "chronic postoperative opioid use"[Title/Abstract] OR "chronic post-operative opioid use"[Title/Abstract] OR "long-term postoperative opioid use"[Title/Abstract] OR "long term postoperative opioid use"[Title/Abstract] OR "new persistent opioid use"[Title/Abstract] OR "extended postoperative opioid use"[Title/Abstract] OR ((persistent[Title/Abstract] OR prolonged[Title/Abstract] OR sustained[Title/Abstract] OR chronic[Title/Abstract] OR "long-term"[Title/Abstract] OR "long term"[Title/Abstract] OR extended[Title/Abstract]) AND (opioid*[Title/Abstract] OR opiate*[Title/Abstract] OR narcotic*[Title/Abstract]) AND (postoperative[Title/Abstract] OR post-operative[Title/Abstract] OR postsurgical[Title/Abstract] OR post-surgical[Title/Abstract] OR perioperative[Title/Abstract] OR peri-operative[Title/Abstract] OR surgery[Title/Abstract] OR surgical[Title/Abstract] OR operative[Title/Abstract])) | 8836 |
| #4 | "Models, Statistical"[Mesh] OR "Forecasting"[Mesh] OR "Machine Learning"[Mesh] OR "Artificial Intelligence"[Mesh] OR "Algorithms"[Mesh] OR "ROC Curve"[Mesh] | 1174963 |
| #5 | "prediction model"[Title/Abstract] OR "prediction models"[Title/Abstract] OR "predictive model"[Title/Abstract] OR "predictive models"[Title/Abstract] OR "risk prediction"[Title/Abstract] OR "risk model"[Title/Abstract] OR "risk models"[Title/Abstract] OR "risk score"[Title/Abstract] OR "risk scores"[Title/Abstract] OR "clinical prediction"[Title/Abstract] OR "prediction tool"[Title/Abstract] OR "predictive tool"[Title/Abstract] OR "risk calculator"[Title/Abstract] OR calculator[Title/Abstract] OR nomogram*[Title/Abstract] OR algorithm*[Title/Abstract] OR "machine learning"[Title/Abstract] OR "artificial intelligence"[Title/Abstract] OR "deep learning"[Title/Abstract] OR "random forest"[Title/Abstract] OR "gradient boosting"[Title/Abstract] OR XGBoost[Title/Abstract] OR "neural network"[Title/Abstract] OR "neural networks"[Title/Abstract] OR "support vector"[Title/Abstract] OR classifier*[Title/Abstract] OR "external validation"[Title/Abstract] OR "model validation"[Title/Abstract] OR calibration[Title/Abstract] OR discrimination[Title/Abstract] OR "C-statistic"[Title/Abstract] OR "C statistic"[Title/Abstract] OR AUROC[Title/Abstract] OR AUC[Title/Abstract] OR Brier[Title/Abstract] | 1403294 |
| #6 | #4 OR #5 | 2147071 |
| #7 | #1 AND #2 AND #3 AND #6 | 443 |

2.Cochrane Library

| **Search number** | **Query** | **Results** |
| --- | --- | --- |
| #1 | MeSH descriptor: [Surgical Procedures, Operative] explode all trees OR MeSH descriptor: [Postoperative Period] explode all trees OR MeSH descriptor: [Perioperative Period] explode all trees OR (surgery OR surgical OR surgeries OR operative OR operation OR operations OR postoperative OR post-operative OR postsurgical OR post-surgical OR perioperative OR peri-operative OR "after surgery" OR "following surgery"):ti,ab,kw | 488808 |
| #2 | MeSH descriptor: [Analgesics, Opioid] explode all trees OR (opioid* OR opiate* OR narcotic* OR oxycodone OR hydrocodone OR morphine OR codeine OR tramadol OR fentanyl OR buprenorphine):ti,ab,kw | 75304 |
| #3 | ("persistent postoperative opioid use" OR "persistent post-operative opioid use" OR "persistent postsurgical opioid use" OR "persistent post-surgical opioid use" OR "prolonged postoperative opioid use" OR "prolonged post-operative opioid use" OR "prolonged postsurgical opioid use" OR "prolonged post-surgical opioid use" OR "prolonged postoperative opioid prescription" OR "prolonged postoperative opioid prescriptions" OR "sustained postoperative opioid use" OR "sustained postoperative opioid prescription" OR "sustained postoperative opioid prescriptions" OR "chronic postoperative opioid use" OR "chronic post-operative opioid use" OR "long-term postoperative opioid use" OR "long term postoperative opioid use" OR "new persistent opioid use" OR "extended postoperative opioid use"):ti,ab,kw OR ((persistent OR prolonged OR sustained OR chronic OR "long-term" OR "long term" OR extended):ti,ab,kw AND (opioid* OR opiate* OR narcotic*):ti,ab,kw AND (postoperative OR post-operative OR postsurgical OR post-surgical OR perioperative OR peri-operative OR surgery OR surgical OR operative):ti,ab,kw) | 5122 |
| #4 | MeSH descriptor: [Models, Statistical] explode all trees OR MeSH descriptor: [Forecasting] explode all trees OR MeSH descriptor: [Machine Learning] explode all trees OR MeSH descriptor: [Artificial Intelligence] explode all trees OR MeSH descriptor: [Algorithms] explode all trees OR MeSH descriptor: [ROC Curve] explode all trees | 34057 |
| #5 | ("prediction model" OR "prediction models" OR "predictive model" OR "predictive models" OR "risk prediction" OR "risk model" OR "risk models" OR "risk score" OR "risk scores" OR "clinical prediction" OR "prediction tool" OR "predictive tool" OR "risk calculator" OR calculator OR nomogram* OR algorithm* OR "machine learning" OR "artificial intelligence" OR "deep learning" OR "random forest" OR "gradient boosting" OR XGBoost OR "neural network" OR "neural networks" OR "support vector" OR classifier* OR "external validation" OR "model validation" OR calibration OR discrimination OR "C-statistic" OR "C statistic" OR AUROC OR AUC OR Brier):ti,ab,kw | 75415 |
| #6 | #4 OR #5 | 99283 |
| #7 | #1 AND #2 AND #3 AND #6 | 135 |

3.Embase

| **Search number** | **Query** | **Results** |
| --- | --- | --- |
| #1 | 'surgery'/exp OR 'postoperative period'/exp OR 'perioperative period'/exp OR surgery:ab,ti OR surgical:ab,ti OR surgeries:ab,ti OR operative:ab,ti OR operation:ab,ti OR operations:ab,ti OR postoperative:ab,ti OR 'post-operative':ab,ti OR postsurgical:ab,ti OR 'post-surgical':ab,ti OR perioperative:ab,ti OR 'peri-operative':ab,ti OR 'after surgery':ab,ti OR 'following surgery':ab,ti | 8653070 |
| #2 | 'opioid analgesic agent'/exp OR opioid*:ab,ti OR opiate*:ab,ti OR narcotic*:ab,ti OR oxycodone:ab,ti OR hydrocodone:ab,ti OR morphine:ab,ti OR codeine:ab,ti OR tramadol:ab,ti OR fentanyl:ab,ti OR buprenorphine:ab,ti | 337665 |
| #3 | 'persistent postoperative opioid use':ab,ti OR 'persistent post-operative opioid use':ab,ti OR 'persistent postsurgical opioid use':ab,ti OR 'persistent post-surgical opioid use':ab,ti OR 'prolonged postoperative opioid use':ab,ti OR 'prolonged post-operative opioid use':ab,ti OR 'prolonged postsurgical opioid use':ab,ti OR 'prolonged post-surgical opioid use':ab,ti OR 'prolonged postoperative opioid prescription':ab,ti OR 'prolonged postoperative opioid prescriptions':ab,ti OR 'sustained postoperative opioid use':ab,ti OR 'sustained postoperative opioid prescription':ab,ti OR 'sustained postoperative opioid prescriptions':ab,ti OR 'chronic postoperative opioid use':ab,ti OR 'chronic post-operative opioid use':ab,ti OR 'long-term postoperative opioid use':ab,ti OR 'long term postoperative opioid use':ab,ti OR 'new persistent opioid use':ab,ti OR 'extended postoperative opioid use':ab,ti OR ((persistent:ab,ti OR prolonged:ab,ti OR sustained:ab,ti OR chronic:ab,ti OR 'long-term':ab,ti OR 'long term':ab,ti OR extended:ab,ti) AND (opioid*:ab,ti OR opiate*:ab,ti OR narcotic*:ab,ti) AND (postoperative:ab,ti OR 'post-operative':ab,ti OR postsurgical:ab,ti OR 'post-surgical':ab,ti OR perioperative:ab,ti OR 'peri-operative':ab,ti OR surgery:ab,ti OR surgical:ab,ti OR operative:ab,ti)) | 17583 |
| #4 | 'statistical model'/exp OR 'prediction'/exp OR 'forecasting'/exp OR 'machine learning'/exp OR 'artificial intelligence'/exp OR 'algorithm'/exp OR 'receiver operating characteristic'/exp | 3096553 |
| #5 | 'prediction model':ab,ti OR 'prediction models':ab,ti OR 'predictive model':ab,ti OR 'predictive models':ab,ti OR 'risk prediction':ab,ti OR 'risk model':ab,ti OR 'risk models':ab,ti OR 'risk score':ab,ti OR 'risk scores':ab,ti OR 'clinical prediction':ab,ti OR 'prediction tool':ab,ti OR 'predictive tool':ab,ti OR 'risk calculator':ab,ti OR calculator:ab,ti OR nomogram*:ab,ti OR algorithm*:ab,ti OR 'machine learning':ab,ti OR 'artificial intelligence':ab,ti OR 'deep learning':ab,ti OR 'random forest':ab,ti OR 'gradient boosting':ab,ti OR XGBoost:ab,ti OR 'neural network':ab,ti OR 'neural networks':ab,ti OR 'support vector':ab,ti OR classifier*:ab,ti OR 'external validation':ab,ti OR 'model validation':ab,ti OR calibration:ab,ti OR discrimination:ab,ti OR 'C-statistic':ab,ti OR 'C statistic':ab,ti OR AUROC:ab,ti OR AUC:ab,ti OR Brier:ab,ti | 1789172 |
| #6 | #4 OR #5 | 3788248 |
| #7 | #1 AND #2 AND #3 AND #6 | 1272 |

4.Web of Science

| **Search number** | **Query** | **Results** |
| --- | --- | --- |
| #1 | TS=(surgery OR surgical OR surgeries OR operative OR operation OR operations OR postoperative OR "post-operative" OR postsurgical OR "post-surgical" OR perioperative OR "peri-operative" OR "after surgery" OR "following surgery") | 9384074 |
| #2 | TS=(opioid* OR opiate* OR narcotic* OR oxycodone OR hydrocodone OR morphine OR codeine OR tramadol OR fentanyl OR buprenorphine) | 395533 |
| #3 | TS=("persistent postoperative opioid use" OR "persistent post-operative opioid use" OR "persistent postsurgical opioid use" OR "persistent post-surgical opioid use" OR "prolonged postoperative opioid use" OR "prolonged post-operative opioid use" OR "prolonged postsurgical opioid use" OR "prolonged post-surgical opioid use" OR "prolonged postoperative opioid prescription" OR "prolonged postoperative opioid prescriptions" OR "sustained postoperative opioid use" OR "sustained postoperative opioid prescription" OR "sustained postoperative opioid prescriptions" OR "chronic postoperative opioid use" OR "chronic post-operative opioid use" OR "long-term postoperative opioid use" OR "long term postoperative opioid use" OR "new persistent opioid use" OR "extended postoperative opioid use" OR ((persistent OR prolonged OR sustained OR chronic OR "long-term" OR "long term" OR extended) AND (opioid* OR opiate* OR narcotic*) AND (postoperative OR "post-operative" OR postsurgical OR "post-surgical" OR perioperative OR "peri-operative" OR surgery OR surgical OR operative))) | 15999 |
| #4 | TS=("prediction model" OR "prediction models" OR "predictive model" OR "predictive models" OR "risk prediction" OR "risk model" OR "risk models" OR "risk score" OR "risk scores" OR "clinical prediction" OR "prediction tool" OR "predictive tool" OR "risk calculator" OR calculator OR nomogram* OR algorithm* OR "machine learning" OR "artificial intelligence" OR "deep learning" OR "random forest" OR "gradient boosting" OR XGBoost OR "neural network" OR "neural networks" OR "support vector" OR classifier* OR "external validation" OR "model validation" OR calibration OR discrimination OR "C-statistic" OR "C statistic" OR AUROC OR AUC OR Brier) | 7439697 |
| #5 | #1 AND #2 AND #3 AND #4 | 501 |

5.Scopus

| **Search number** | **Query** | **Results** |
| --- | --- | --- |
| #1 | TITLE-ABS-KEY(surgery OR surgical OR surgeries OR operative OR operation OR operations OR postoperative OR "post-operative" OR postsurgical OR "post-surgical" OR perioperative OR "peri-operative" OR "after surgery" OR "following surgery") | 7793930 |
| #2 | TITLE-ABS-KEY(opioid* OR opiate* OR narcotic* OR oxycodone OR hydrocodone OR morphine OR codeine OR tramadol OR fentanyl OR buprenorphine) | 478531 |
| #3 | TITLE-ABS-KEY("persistent postoperative opioid use" OR "persistent post-operative opioid use" OR "persistent postsurgical opioid use" OR "persistent post-surgical opioid use" OR "prolonged postoperative opioid use" OR "prolonged post-operative opioid use" OR "prolonged postsurgical opioid use" OR "prolonged post-surgical opioid use" OR "prolonged postoperative opioid prescription" OR "prolonged postoperative opioid prescriptions" OR "sustained postoperative opioid use" OR "sustained postoperative opioid prescription" OR "sustained postoperative opioid prescriptions" OR "chronic postoperative opioid use" OR "chronic post-operative opioid use" OR "long-term postoperative opioid use" OR "long term postoperative opioid use" OR "new persistent opioid use" OR "extended postoperative opioid use" OR ((persistent OR prolonged OR sustained OR chronic OR "long-term" OR "long term" OR extended) AND (opioid* OR opiate* OR narcotic*) AND (postoperative OR "post-operative" OR postsurgical OR "post-surgical" OR perioperative OR "peri-operative" OR surgery OR surgical OR operative))) | 16916 |
| #4 | TITLE-ABS-KEY("prediction model" OR "prediction models" OR "predictive model" OR "predictive models" OR "risk prediction" OR "risk model" OR "risk models" OR "risk score" OR "risk scores" OR "clinical prediction" OR "prediction tool" OR "predictive tool" OR "risk calculator" OR calculator OR nomogram* OR algorithm* OR "machine learning" OR "artificial intelligence" OR "deep learning" OR "random forest" OR "gradient boosting" OR XGBoost OR "neural network" OR "neural networks" OR "support vector" OR classifier* OR "external validation" OR "model validation" OR calibration OR discrimination OR "C-statistic" OR "C statistic" OR AUROC OR AUC OR Brier) | 8674800 |
| #5 | #1 AND #2 AND #3 AND #4 | 587 |

# Table S2. Predictor selection methods and candidate/final predictors.

| **Study** | **Predictor-selection / modelling approach** | **Predictors/features reported** | **Main predictor domains reported** |
| --- | --- | --- | --- |
| Anderson AB 2020 | Boruta feature selection; GBM tuning/final model. | 7 | Prior opioid exposure/dose; demographics; geographic and military context; prescribing-site features. |
| Bateman Bt 2016 | Multivariable logistic regression; no automated selection reported. | 8 | Demographics; substance/tobacco use; pain diagnoses; mental-health medication. |
| Castle 2023 | Cox/logistic modelling with naive Bayes variable importance. | Top 5 naive Bayes importance features; multivariable model included 9 covariate terms | Demographics; BMI; prior opioid exposure; procedure and revision features; cartilage/pathology features. |
| Chaudhary Ma 2019 | Backward stepwise regression. | 9 | Demographics; discharge and length of stay; socioeconomic/insurance proxy; procedure category; mental health; prior opioid use. |
| Chen 2025 | External validation of ACDF SORG-MLA; no new selection. | 9 | Demographics; procedure complexity; insurance; tobacco use; medication history; preoperative opioid duration. |
| Chen Sf 2023 | External validation of lumbar SORG-MLA; no new selection. | 13 | Demographics; procedure complexity/approach; insurance; laboratory values; tobacco/alcohol use; medication history; mental health; diagnosis. |
| Crawford Am 2023 | External validation of SOS score; no new selection. | 10 | Demographics; discharge and length of stay; socioeconomic/insurance proxy; procedure category; diabetes; mental health; prior opioid use. |
| Ekman E 2026 | Multivariable regression; no automated selection reported. | 8 | Demographics; trauma characteristics; season; length of stay; socioeconomic status; mental health; preoperative opioid use. |
| Gabriel 2022 | Balanced ensemble ML; variable importance reported. | Top 7 importance features reported; no formal retained-feature subset stated | Early postoperative opioid use; discharge prescribing; demographics/BMI; prior opioid use; length of stay; procedure type. |
| Gessner Kh 2025 | Multivariable logistic regression with restricted candidate set. | 18 | Demographics; tobacco use; pain diagnoses/scores; medication history; mental health; local socioeconomic and opioid-environment measures. |
| Giladi 2023 | Ensemble feature ranking plus SHAP. | Feature-selected subset; exact retained n not reported, Top/protective predictors reported | Pain/function/PROs; race; tobacco/substance/alcohol use; cancer; mental health; income. |
| Grazal 2022 | Boruta feature selection. | 5 | Demographics; preoperative and postoperative opioid use; mental health; substance use. |
| Guattery J 2025 | Predefined inputs plus SHAP/LIME explainability. | 23 input variables; top SHAP/LIME features reported | Demographics; BMI; tobacco use; mental health; comorbidity; procedure indicators; preoperative opioid fills. |
| Harris 2020 | Multivariable logistic regression; no automated selection reported. | 10 | Prior opioid use; substance/tobacco use; mental health; geography; readmission; cardiopulmonary comorbidity; hypertension. |
| Hur 2021 | High-dimensional preprocessing with SVM/XGBoost; permutation importance. | 4,989 input features; top feature groups reported | High-dimensional claims features; surgery type; opioid/prescription features; geography; medication history; tobacco use; pain diagnoses; education. |
| Karhade 2019 | External validation of SOS score; no new selection. | 10 | Demographics; discharge and length of stay; socioeconomic/insurance proxy; procedure category; diabetes; mental health; prior opioid use. |
| Karhade Av 2020 | External validation/comparison of SOS score; no new selection. | 10 | Demographics; discharge and length of stay; socioeconomic/insurance proxy; procedure category; diabetes; mental health; prior opioid use. |
| Kim J 2025 | Candidate variables entered; SHAP importance. | 28 | Demographics; comorbidity; psychological disorders; substance use; medication history; surgery type; length of stay; perioperative opioid dose. |
| Kunze 2021 | Recursive feature elimination and importance ranking. | 7 | Demographics/BMI; pain/function scores; workers' compensation; activity/sport participation; race. |
| Lu Y 2022 | RF recursive feature elimination. | 11 | Pain/function/PROs; demographics; symptom duration; perioperative opioid dose; previous injections/nerve blocks; exercise frequency. |
| Naderalvojoud 2025 | External validation/fairness assessment of pretrained LASSO model. | Exact final retained count NR | High-dimensional EHR/claims domains: conditions, procedures, measurements and drugs; exact retained predictors not reported. |
| Naderalvojoud 2023 | OHDSI covariate extraction, PNF filtering and ensemble ML. | 35,864 extracted covariates; 3,418 PNF-selected relevant covariates; final retained count NR | High-dimensional OHDSI EHR/claims covariates; final named retained predictors not reported. |
| Nicholson Ad 2019 | Multivariable logistic regression/nomogram; no automated selection reported. | 10 | Prior opioid use; insurance; BMI; procedure type; psychiatric disorder; smoking; cardiopulmonary, neurologic and gastrointestinal/hepatobiliary comorbidity. |
| Shipp Mm 2022 | LASSO followed by reduced multivariable logistic regression. | 10 model covariate domains | Demographics/BMI; surgery and trauma/pathology type; substance use; pain/function/PROs. |
| Simpson S 2024 | Supervised ML; procedure-specific permutation importance. | Procedure-specific PFI variables reported; full retained/input feature count NR | Procedure-specific ML importance features; length of stay; demographics; operative time; smoking; laboratory values; kidney disease; insurance. |
| Singh K 2022 | Expert-preselected restricted logistic model. | 10 | Demographics; comorbidity; preoperative opioid fills; prescription features; pain/function/PROs; psychosocial measures. |
| Soley 2024 | ML with SMOTE/tuning; SHAP for stacked model. | Exact input feature count NR; SHAP important features reported across five input domains | Demographics/socioeconomic status; medical history; laboratory values; wearable activity/sleep data; surgery type; prior opioid use. |
| Soley 2025 | EHR/wearable/genetic ML; SHAP and fairness preprocessing. | 34 input features (19 EHR/wearable + 15 SNPs); SHAP top features reported | EHR/wearable features; genetic variants; activity/sleep; surgery type; medical history; laboratory values; prior opioid use; mental health. |
| Sun M 2024 | Stepwise multivariable model. | 15 | Demographics; residence; surgery/anesthesia features; preoperative analgesic/opioid use; mental health; pressure ulcer; comorbidity. |
| Tsai Cc 2023 | External validation of TKA SORG-MLA; no new selection. | 9 | Demographics; prior opioid use; marital status; diabetes; medication history. |
| Tudorache Ym 2026 | Backward elimination. | 8 | Demographics; fracture type; postoperative mobility; marital status; preoperative medication use; surgical delay; length of stay. |
| Ward A 2021 | LASSO/RF/GBM/XGBoost with variable pruning and permutation importance. | 109 retained variables for day-of-surgery model; 136 for 14-day after-surgery model | High-dimensional claims/EHR features; preoperative opioid use; perioperative/postoperative opioid supply and MME; procedure type; socioeconomic context. |
| Yeh Ph 2026 | LASSO-Elastic Net logistic regression. | 28 | Demographics; urbanisation; baseline diagnoses/comorbidity; medication history; disease duration; hospital level; length of stay; anesthesia and postdischarge medications. |
| Yen 2022 | External validation of lumbar disc herniation SORG-MLA; no new selection. | 45 | Demographics; spine procedure features; laboratory values; socioeconomic/neighbourhood factors; preoperative opioid category; medication history; substance use; comorbidity. |
| Zhang Y 2020 | Model comparison; final full logistic regression. | 14 | Opioid supply and dose-change patterns; number of prescriptions; total MME; fusion surgery; comorbidity; demographics; mental health. |

**Abbreviations:** EHR, electronic health record; GBM, gradient boosting machine; LASSO, least absolute shrinkage and selection operator; ML, machine learning; MGI, Michigan Genomics Initiative; MME, morphine milligram equivalents; NR, not reported; OHDSI, Observational Health Data Sciences and Informatics; PNF, patient-level prediction framework; PRO, patient-reported outcome; RF, random forest; SHAP, Shapley additive explanations; SORG-MLA, Skeletal Oncology Research Group machine learning algorithm; SVM, support vector machine.

**Supplementary Table S3. Exploratory univariable meta-regression of study-level AUROC**

| **Moderator** | **Comparison (n)** | **Beta, logit AUROC (95% CI)** | ***P*** | **Omnibus *P*** | **Residual tau^2^** | **Residual I^2^** | **FDR q** |
| --- | --- | --- | --- | --- | --- | --- | --- |
| Validation status | External/temporal validation (n=9) vs Development/internal validation (n=26) | -0.343 (-0.901 to 0.214) | 0.219 | 0.219 | 0.388 | 99.4 | 0.438 |
| Baseline opioid status | Opioid-naive (n=7) vs Mixed/prior use included (n=28) | -0.532 (-1.115 to 0.050) | 0.072 | 0.072 | 0.351 | 99.8 | 0.360 |
| Prediction timing | Intraop/discharge/in-hospital (n=11) vs Preoperative only (n=19) | 0.356 (-0.207 to 0.919) | 0.207 | 0.401 | 0.415 | 99.7 | 0.573 |
|  | Postoperative-updated (n=5) vs Preoperative only (n=19) | -0.036 (-0.764 to 0.691) | 0.919 |  |  |  |  |
| Surgical/procedure domain | Other/non-ortho (n=6) vs Orthopedic/extremity (n=14) | 0.109 (-0.641 to 0.859) | 0.768 | 0.991 | 0.446 | 99.8 | 0.991 |
|  | Mixed surgery (n=8) vs Orthopedic/extremity (n=14) | 0.055 (-0.625 to 0.734) | 0.870 |  |  |  |  |
|  | Spine/neurosurgery (n=7) vs Orthopedic/extremity (n=14) | 0.082 (-0.640 to 0.803) | 0.819 |  |  |  |  |
| Model class | Machine learning/penalized model (n=18) vs Conventional regression (n=7) | 0.499 (-0.141 to 1.138) | 0.122 | 0.343 | 0.389 | 99.9 | 0.572 |
|  | Other/unclear (n=2) vs Conventional regression (n=7) | 0.736 (-0.393 to 1.865) | 0.193 |  |  |  |  |
|  | Risk score/calculator/existing model (n=8) vs Conventional regression (n=7) | 0.212 (-0.529 to 0.953) | 0.564 |  |  |  |  |
| Outcome window | Unclear/mixed follow-up window (n=4) vs Long window: >=6 months or 6-12 months (n=22) | -0.550 (-1.297 to 0.197) | 0.144 | 0.156 | 0.351 | 98.5 | 0.420 |
|  | Early extended window: >30 days to <90 days (n=3) vs Long window: >=6 months or 6-12 months (n=22) | -0.353 (-1.243 to 0.537) | 0.424 |  |  |  |  |
|  | Intermediate window: around 90-180 days (n=6) vs Long window: >=6 months or 6-12 months (n=22) | -0.632 (-1.264 to 0.000) | 0.050 |  |  |  |  |
| Operational outcome | Any fill/refill/use at follow-up definition (n=17) vs Repeated or sustained prescription/supply definition (n=15) | -0.030 (-0.563 to 0.502) | 0.908 | 0.743 | 0.420 | 99.8 | 0.826 |
|  | Other or unclear operational definition (n=3) vs Repeated or sustained prescription/supply definition (n=15) | -0.349 (-1.281 to 0.582) | 0.450 |  |  |  |  |
| Calibration reporting | Calibration reported (n=22) vs Calibration not reported (n=13) | -0.549 (-1.025 to -0.072) | 0.025 | 0.025 | 0.348 | 99.6 | 0.253 |
| DCA reporting | DCA reported (n=11) vs DCA not reported (n=24) | -0.361 (-0.881 to 0.160) | 0.168 | 0.168 | 0.383 | 99.4 | 0.420 |
| PROBAST overall | Low (n=6) vs Unclear (n=20) | -0.328 (-1.013 to 0.358) | 0.338 | 0.517 | 0.415 | 99.8 | 0.646 |
|  | High (n=9) vs Unclear (n=20) | 0.106 (-0.498 to 0.710) | 0.722 |  |  |  |  |

# Table S4. PROBAST risk-of-bias assessment.

| **Study** | **Participants** | **Predictors** | **Outcome** | **Analysis** | **Overall risk of bias** |
| --- | --- | --- | --- | --- | --- |
| Anderson AB 2020 | Low | Low | Low | Low | Low |
| Bateman Bt 2016 | Low | Low | Low | High | High |
| Castle 2023 | Low | Low | Low | High | High |
| Chaudhary Ma 2019 | Low | Low | Low | High | High |
| Chen 2025 | Low | Low | Low | Low | Low |
| Chen Sf 2023 | Low | Low | Low | Low | Low |
| Crawford Am 2023 | Low | Low | Low | Unclear | Unclear |
| Ekman E 2026 | Low | Low | Low | Unclear | Unclear |
| Gabriel 2022 | Low | Unclear | Low | High | High |
| Gessner Kh 2025 | Low | Low | Low | Unclear | Unclear |
| Giladi 2023 | Low | Unclear | Low | Unclear | Unclear |
| Grazal 2022 | Low | Low | Low | Unclear | Unclear |
| Guattery J 2025 | Low | Low | Low | High | High |
| Harris 2020 | Low | Low | Low | High | High |
| Hur 2021 | Low | Unclear | Low | High | High |
| Karhade 2019 | Low | Low | Low | Unclear | Unclear |
| Karhade Av 2020 | Low | Low | Low | Unclear | Unclear |
| Kim J 2025 | Low | Low | Low | Unclear | Unclear |
| Kunze 2021 | Low | Low | Low | Unclear | Unclear |
| Lu Y 2022 | Low | Low | Low | Unclear | Unclear |
| Naderalvojoud 2025 | Low | Low | Low | Unclear | Unclear |
| Naderalvojoud 2023 | Low | Unclear | Low | Unclear | Unclear |
| Nicholson Ad 2019 | Low | Low | Low | Unclear | Unclear |
| Shipp Mm 2022 | Low | Low | Low | Unclear | Unclear |
| Simpson S 2024 | Low | Unclear | Low | Unclear | Unclear |
| Singh K 2022 | Low | Low | Low | Unclear | Unclear |
| Soley 2024 | Low | Low | Low | Unclear | Unclear |
| Soley 2025 | Low | Unclear | Low | Unclear | Unclear |
| Sun M 2024 | Low | Low | Low | High | High |
| Tsai Cc 2023 | Low | Low | Low | Low | Low |
| Tudorache Ym 2026 | Low | Low | Low | Low | Low |
| Ward A 2021 | Low | Unclear | Low | High | High |
| Yeh Ph 2026 | Low | Low | Low | Unclear | Unclear |
| Yen 2022 | Low | Low | Low | Low | Low |
| Zhang Y 2020 | Low | Low | Low | Unclear | Unclear |

# Table S5. PROBAST applicability concern assessment.

| **Study** | **Participants** | **Predictors/timing** | **Outcome** | **Overall applicability concern** |
| --- | --- | --- | --- | --- |
| Anderson AB 2020 | Low | Low | Low | Low |
| Bateman Bt 2016 | Low | Low | Low | Low |
| Castle 2023 | Low | Low | Low | Low |
| Chaudhary Ma 2019 | Low | Low | Low | Low |
| Chen 2025 | Low | Low | Low | Low |
| Chen Sf 2023 | Low | Low | Low | Low |
| Crawford Am 2023 | Low | Low | Low | Low |
| Ekman E 2026 | Low | Low | Low | Low |
| Gabriel 2022 | Low | Low | Low | Low |
| Gessner Kh 2025 | Low | Low | Low | Low |
| Giladi 2023 | Low | Low | Low | Low |
| Grazal 2022 | Low | Low | Low | Low |
| Guattery J 2025 | Low | Low | Low | Low |
| Harris 2020 | Low | Low | Low | Low |
| Hur 2021 | Low | Low | Low | Low |
| Karhade 2019 | Low | Low | Low | Low |
| Karhade Av 2020 | Low | Low | Low | Low |
| Kim J 2025 | Low | Low | Low | Low |
| Kunze 2021 | Low | Low | Low | Low |
| Lu Y 2022 | Low | Low | Low | Low |
| Naderalvojoud 2025 | Low | Low | Low | Low |
| Naderalvojoud 2023 | Low | Low | Low | Low |
| Nicholson Ad 2019 | Low | Low | Low | Low |
| Shipp Mm 2022 | Low | Low | Low | Low |
| Simpson S 2024 | Low | Low | Low | Low |
| Singh K 2022 | Low | Low | Low | Low |
| Soley 2024 | Low | Low | Low | Low |
| Soley 2025 | Low | Low | Low | Low |
| Sun M 2024 | Low | Low | Low | Low |
| Tsai Cc 2023 | Low | Low | Low | Low |
| Tudorache Ym 2026 | Low | Low | Low | Low |
| Ward A 2021 | Low | Low | Low | Low |
| Yeh Ph 2026 | Low | Low | Low | Low |
| Yen 2022 | Low | Low | Low | Low |
| Zhang Y 2020 | Low | Low | Low | Low |

# Table S6. Predefined translational-evidence benchmarks.

| **Criterion** | ***n*** | **%** |
| --- | --- | --- |
| External/temporal validation | 9 | 25.7 |
| Calibration reported | 22 | 62.9 |
| Decision curve analysis reported | 11 | 31.4 |
| Low PROBAST overall | 6 | 17.1 |
| External/temporal validation + calibration + DCA | 5 | 14.3 |
| All four predefined translational-evidence benchmarks | 4 | 11.4 |
